# Prediction of tumour grade and survival outcome using pre-treatment PET- and MRI-derived imaging features in patients with resectable pancreatic ductal adenocarcinoma

**DOI:** 10.1007/s00330-020-07191-z

**Published:** 2020-08-26

**Authors:** Vincent Dunet, Nermin Halkic, Christine Sempoux, Nicolas Demartines, Michael Montemurro, John O. Prior, Sabine Schmidt

**Affiliations:** 1 Department of Diagnostic and Interventional Radiology, Lausanne University Hospital and University of Lausanne, Lausanne, Switzerland; 2 Department of Visceral Surgery, Lausanne University Hospital and University of Lausanne, Lausanne, Switzerland; 3 Institute of Pathology, Lausanne University Hospital and University of Lausanne, Lausanne, Switzerland; 4 Department of Oncology, Lausanne University Hospital and University of Lausanne, Lausanne, Switzerland; 5 Department of Nuclear Medicine and Molecular Imaging, Lausanne University Hospital and University of Lausanne, Rue du Bugnon 46, CH-1011 Lausanne, Switzerland

**Keywords:** Carcinoma, Pancreatic ductal, Diffusion magnetic resonance imaging, Fluorodeoxyglucose F-18, Progression-free survival, Patient outcome assessment

## Abstract

**Objectives:**

To perform a correlation analysis between histopathology and imaging in patients with previously untreated pancreatic ductal adenocarcinoma (PDAC) and to determine the prognostic values of clinical, histological, and imaging parameters regarding overall survival (OS), disease-specific survival (DSS), and progression-free survival (PFS).

**Methods:**

This single-centre study prospectively included 61 patients (32 males; median age, 68.0 years [IQR, 63.0–75.0 years]) with histologically confirmed PDAC and following surgical resection who preoperatively underwent ^18^F-FDG PET/CT and DW-MRI. On whole lesions, we measured, using a 42% SUV_max_ threshold volume of interest (VOI), the following quantitative parameters: mean and maximum standardised uptake values (SUV_mean_ and SUV_max_), total lesion glycolysis (TLG), metabolic tumour volume (MTV), mean and minimum apparent diffusion coefficient (ADC_mean_ and ADC_min_), diffusion total volume (DTV), and MTV/ADC_min_ ratio. Spearman’s correlation analysis was performed to assess relationships between these markers and histopathological findings from surgical specimens (stage; grade; resection quality; and vascular, perineural, and lymphatic invasion). Kaplan-Meier and Cox hazard ratio methods were used to evaluate the impacts of imaging parameters on OS (*n* = 41), DSS (*n* = 36), and PFS (*n* = 41).

**Results:**

Inverse correlations between ADC_min_ and SUV_max_ (rho = − 0.34; *p* = 0.0071), and between SUV_mean_ and ADC_mean_ (rho = − 0.29; *p* = 0.026) were identified. ADC_min_ was inversely correlated with tumour grade (rho = − 0.40; *p* = 0.0015). MTV was an independent predictive factor for OS and DSS, while DTV was an independent predictive factor for PFS.

**Conclusion:**

In previously untreated PDAC, ADC and SUV values are correlated. Combining PET-MRI metrics may help predict PDAC grade and patients’ survival.

**Key Points:**

• *Minimum apparent diffusion coefficient derived from DW-MRI inversely correlates with tumour grade in pancreatic ductal adenocarcinoma*.

• *In pancreatic ductal adenocarcinoma, metabolic tumour volume has been confirmed as a predictive factor for patients’ overall survival and disease-specific survival*.

• *Combining PET and MRI metrics may help predict grade and patients’ survival in pancreatic ductal adenocarcinoma*.

**Electronic supplementary material:**

The online version of this article (10.1007/s00330-020-07191-z) contains supplementary material, which is available to authorized users.

## Introduction

Pancreatic cancer is the second most common digestive cancer and accounts for over 441,000 deaths worldwide [1]. Pancreatic ductal adenocarcinoma (PDAC) is the most frequent pancreatic malignancy and shows a rising incidence [1] with a poor prognosis despite recent advancements in management. Indeed, patients with PDAC have a 5-year survival rate of only 4% [2]. Notably, pancreatic cancer exhibits a poor response to most chemotherapeutic agents, such that surgery is the only curative treatment.

Accurate preoperative staging is required to select patients who are eligible for surgical resection with negative margins [3]. Magnetic resonance imaging (MRI) can identify patients who are unresectable due to liver metastasis or arterial encasement, while fluorine-18 fluorodeoxyglucose (^18^F-FDG) positron emission tomography coupled with computed tomography (PET/CT) can identify patients with distant metastatic disease. ^18^F-FDG PET/CT and MRI have proven useful for preoperative differentiation between benign and malignant pancreatic disease [4–7]. Among patients with resectable disease, quantitative imaging metrics can help identifying patients likely to have poor outcomes [8–11], similarly to histological characteristics obtained from surgical specimens [12]. However, there is a paucity of evidence on the correlation between quantitative imaging metrics and pathological features [6–8, 13–15]. Moreover, we wondered if initial ^18^F-FDG PET/CT and MRI parameters, as well as any clinical and histological parameters, were useful for predicting patient survival in a single population.

The present study aimed to perform a correlation analysis between histopathology and imaging in patients with untreated PDAC and to determine the prognostic value of clinical, histological, and imaging-related parameters in terms of overall survival (OS), disease-specific survival (DSS), and progression-free survival (PFS).

## Methods

### Study population

This study was designed as a transparent reporting of a multivariable prediction model for individual prognosis or diagnosis (TRIPOD) type 1a study to assess the potential benefit of pre-treatment PET- and MRI-derived imaging features in patients with operable PDAC [16]. The TRIPOD checklist is provided in Supplementary Table 1.

From July 2008 to July 2017, all patients with suspected PDAC were prospectively and consecutively enrolled, provided they were not previously treated. The inclusion criteria were age > 18 years, suspected PDAC without previous treatment, and planned curative surgical resection at our hospital. Exclusion criteria were pregnancy, contraindications to MRI, detection of unresectable tumour on preoperative imaging or during surgery, and non-PDAC lesions on histology. All patients underwent both ^18^F-FDG PET/CT and DW-MRI examination before surgery. Operated patients with confirmed PDAC based on surgical specimens, and with complete imaging and histopathological datasets, were included in a radiopathological correlation analysis and in the follow-up study. All participants gave their written informed consent 48 h prior to inclusion in the study. The protocol was approved by our institutional review board and local research ethics committee (study #119/08).

### Imaging protocols

The ^18^F-FDG PET/CT examinations were performed using a Discovery LS scanner until 08/2011 (*n* = 34), and thereafter with a Discovery 690 PET/CT scanner (GE Healthcare; *n* = 27). Before examination, patients fasted for > 6 h, and blood glucose level was verified to be ≤ 8.3 mmol/L prior to ^18^F-FDG administration. Patients were injected with ^18^F-FDG: 5.5 MBq/kg until 08/2011, and thereafter with 3.5 MBq/kg or 309 ± 81 MBq (range: 158–488 MBq). At 68 ± 11 min (range: 50–95 min) after injection, we performed PET acquisition from the vertex to mid-thigh: two-dimensional mode with 6–7 steps of 3–5 min for the Discovery LS or three-dimensional mode with 8–9 steps of 2 min for the Discovery 690 (mean duration: 20 ± 5 min; range: 16–35 min). All PET acquisition and reconstruction parameters are displayed in Supplementary Table 2. For attenuation correction, the PET acquisition followed an unenhanced MDCT acquisition from the vertex to mid-thigh: 140 kV, 80 mA, pitch 1.5, 0.5 s/rotation, 5-mm slice thickness until 08/2011; and 120 keV, 80–200 AutomA/SmartmA, pitch 1.375, and 3.3-mm slice thickness thereafter. Standardised uptake values (SUV) were corrected for body mass [17, 18]. Between scanners, stability of PET data of our centre was ensured by biannually acquiring a uniform phantom over the study period [19].

MR data were acquired using a 1.5-T scanner (*n* = 33, Symphony or Aera; Siemens Healthcare) or a 3.0-T scanner (*n* = 28; Skyra, Prisma, or Verio, Siemens Healthcare) with an 18-channel phased-array body coil covering the upper abdomen, combined with a 32-channel spine coil. MR acquisition was performed as described in Supplementary Table 3 and included a transverse single-shot spin-echo echo-planar DWI sequence in three orthogonal directions (frequency-encoding, phase-encoding, and slice selection) with three *b*-values ranging from 50 to 800 s/mm^2^ in increasing order. Pixel-to-pixel ADC maps were generated from DW-MRI sequences using Siemens software.

### Imaging analysis

^18^F-FDG PET and DW-MR data were analysed by one reader having 9 years of experience in abdominal imaging (nuclear medicine and radiology) on the same workstation (Advantage Workstation 4.6, GE Healthcare). Quantitative parameter values were measured over the pancreatic lesions. To assess the whole tumour, quantitative parameters using volume of interest (VOI), embedding the entire pancreatic lesions, were evaluated.

On unenhanced ^18^F-FDG PET/CT images, a 42% SUV_max_ threshold VOI around each visible pancreatic lesion was drawn [20, 21]. On a per-lesion basis, we recorded the SUV_max_, SUV_mean_, metabolic tumour volume (MTV) in millilitres, and total lesion glycolysis (TLG = SUV_mean_ × MTV).

On DW-MR images, we manually drew a VOI encompassing the whole lesion on the intermediate of the three *b*-values. We then copied and pasted this VOI on the ADC maps to measure the corresponding mean and minimum ADCs (ADC_mean_ and ADC_min_). Compared with the two-dimensional ROI method, the VOI method was preferred because it demonstrated good inter-observer reproducibility for both ADC_mean_ and ADC_min_ while containing the areas of the tumour with the highest cellular density in the tumour [22]. The diffusion tumour volume (DTV) was defined as the VOI volume in millilitres. Finally, we calculated the MTV/ADC_min_ ratio [10].

### Histopathological analysis

All surgical specimens were consecutively subjected to macroscopical and microscopical analyses by a board-certified pathologist who specialised in digestive oncology with a practical experience of 20 years. An internal standardised protocol was used to assess pTNM, grade (1, well-differentiated; 2, moderately differentiated; 3, poorly differentiated; and 4, undifferentiated), resection quality (R0 or R1), vascular invasion (V score of 0 if absent, and 1 if present), perineural invasion (Pn score of 0 if absent, or 1 if present), and lymphatic invasion (L score of 0 if absent, or 1 if present). Over the study time period, pTNM staging changed; thus, the pathology reports were reviewed and adapted according to the most recent UICC classification [23].

### Follow-up and outcome

Patients with PDAC verified from surgical specimens were included in the follow-up part. OS was defined as the time from surgery until death from any cause. DSS was defined as the time from surgery until PDAC-related death (with death not related to PDAC being censored). PFS was defined as the time from surgery until progression of the oncological disease (with death not related to PDAC and arising before progression being censored). Follow-up data as of July 2018 were collected from patients’ medical history available in the hospital information system, and using a questionnaire sent to the patients’ referring physicians.

### Statistical analysis

All statistical analyses were performed using Stata 15.1 software (StataCorp). Continuous variables are presented as median [interquartile range (IQR)] and categorical variables as number and/or percentage (%) and were compared using the Wilcoxon rank-sum test and the Fisher’s exact test, respectively. For the radiopathological correlation analysis (*n* = 61 patients), we calculated the Spearman’s correlation coefficient to assess the relationships between quantitative parameter values (SUV_max_, SUV_mean_, MTV, TLG, ADC_min_, ADC_mean_, DTV, and MTV/ADC_min_ ratio) and histopathological findings (stage; grade; resection; and vascular, perineural, and lymphatic invasion). Imaging metrics were compared between patients scanned with different PET and MRI devices to evaluate potential bias.

For survival analysis (*n* = 41 patients), each continuous variable was dichotomised using optimal cutoff values determined by the Youden method on receiver operating characteristic (ROC) curves. Outcome was analysed using the Kaplan-Meier method with event-free survival curves and compared using the log-rank test. Univariate and stepwise multivariate analyses were performed to identify independent predictors of death (OS and DSS) and progression (PFS) using the Cox proportional hazard ratio model with the clinical parameters (sex, stage), imaging parameters, magnetic resonance strength field, and PET type. Stepwise multivariate analysis was performed taking into account variables with a *p* < 0.10 for forward and *p* < 0.05 for backward selection. A *p* value of < 0.05 was considered to indicate statistical significance. Bonferroni-corrected *p* values were calculated for multivariate analysis and are noted in table footnotes.

## Results

### Study population’s characteristics

Of the initially enrolled 87 patients, 6 were excluded from radiopathological correlation analysis because they were ultimately considered unresectable, and 20 were excluded because they had non-PDAC lesions. The radiopathological correlation analysis included 61 patients (32 males; mean age, 68 years [IQR, 63–75 years]).

All 61 included patients underwent both ^18^F-FDG PET/CT and DW-MR examinations within 3 days [IQR, 1–7 days], followed by surgical resection within 10 days [IQR, 6–21 days]. Of these patients, 48 underwent cephalic duodenopancreatectomy, 2 total pancreatectomy, and 11 distal pancreatectomy. Additionally, 52 patients underwent adjuvant chemotherapy with gemcitabine (*n* = 46), gemcitabine-capecitabine (*n* = 3), FOLFIRINOX (*n* = 2), or Xeloda (*n* = 1).

**Fig. 1:**
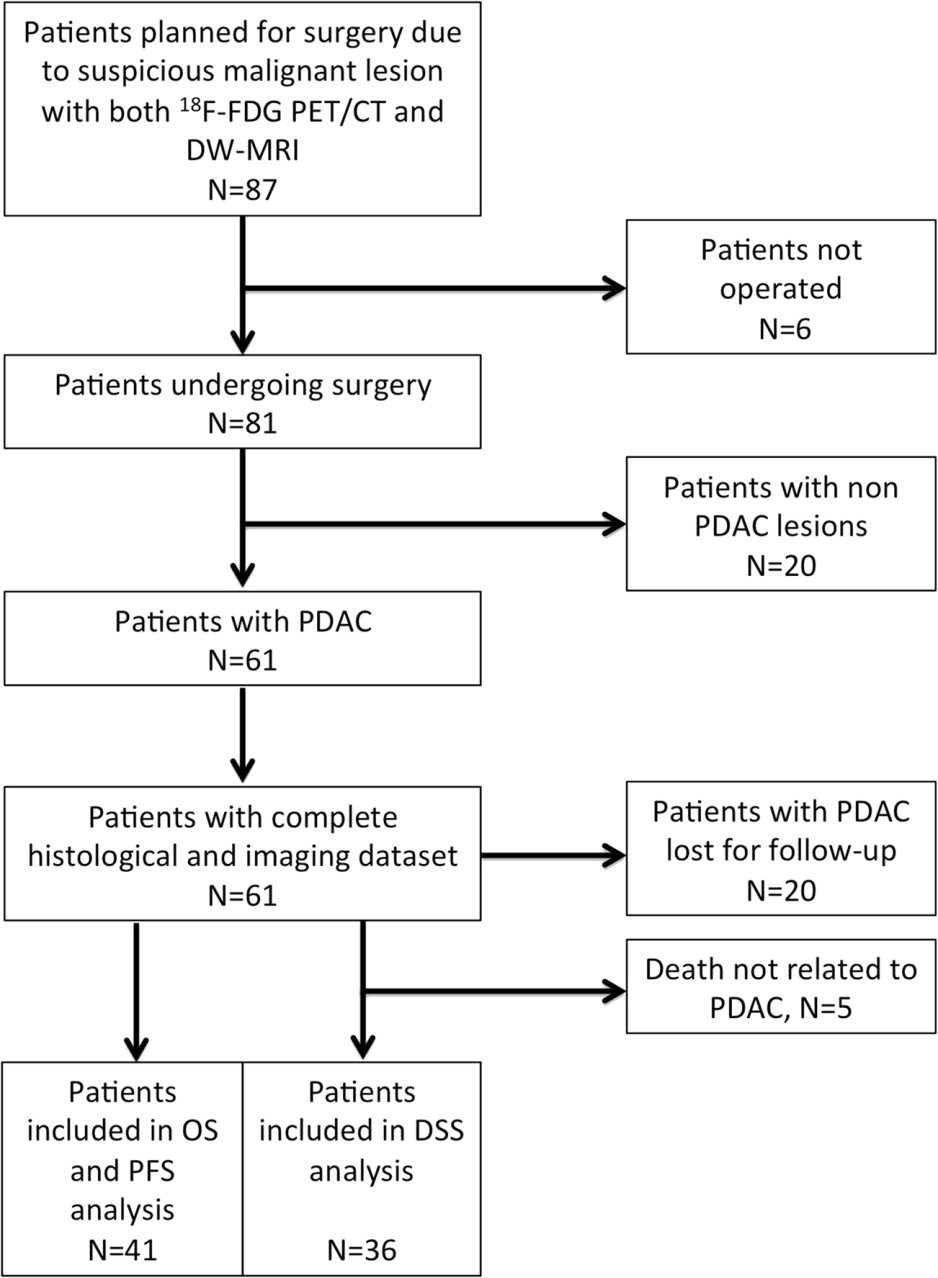
Study flowchart showing inclusion and exclusion criteria

Twenty patients were lost for follow-up evaluation, and five patients died from non-PDAC-related causes and were excluded from the DSS analysis (Fig. 1). Thus, the survival analysis included a total of 41 patients (21 males; median age, 68 years [IQR, 61–75 years]) (Table 1).

**Table 1:** Patients’ and tumours’ characteristics (*n* = 61)

Variables	PDAC, *N* = 61
Age (years)	68.0 [63.0–75.0]
Sex, male/female	32/29
Tumour stage, 1a/1b/2a/2b/3/4	1/3/4/29/21/3
Tumour grade, 1/2/3	9/36/16
Resection, 0/1	30/31
Perineural invasion, 0/1	2/59
Vascular invasion, 0/1	12/49
Lymphatic invasion, 0/1	14/47
SUV_max_ (g/mL)	7.6 [5.7–8.9]
SUV_mean_ (g/mL)	4.2 [3.1–5.2]
MTV (cm^3^)	9.4 [6.0–15.6]
TLG (g·cm^3^/mL)	44.7 [21.5–67.3]
ADC_min_ (10^−6^ mm^2^/s)	718 [600–950]
ADC_mean_ (10^−6^ mm^2^/s)	1430 [1258–1630]
DTV (cm^3^)	8.4 [5.8–14.9]
MTV/ADC_min_ ratio (×10^3^)	14.9 [7.2–22.8]

*ADC*, apparent diffusion coefficient; *DTV*, diffusion tumour volume; *MTV*, metabolic tumour volume; *PDAC*, pancreatic ductal adenocarcinoma; *SUV*, standardised uptake value; *TLG*, total lesion glycolysis. The right column indicates numbers or median [interquartile range]

### Patients characteristics, tumour parameters, and correlation between histopathology and imaging

**Fig. 2:**
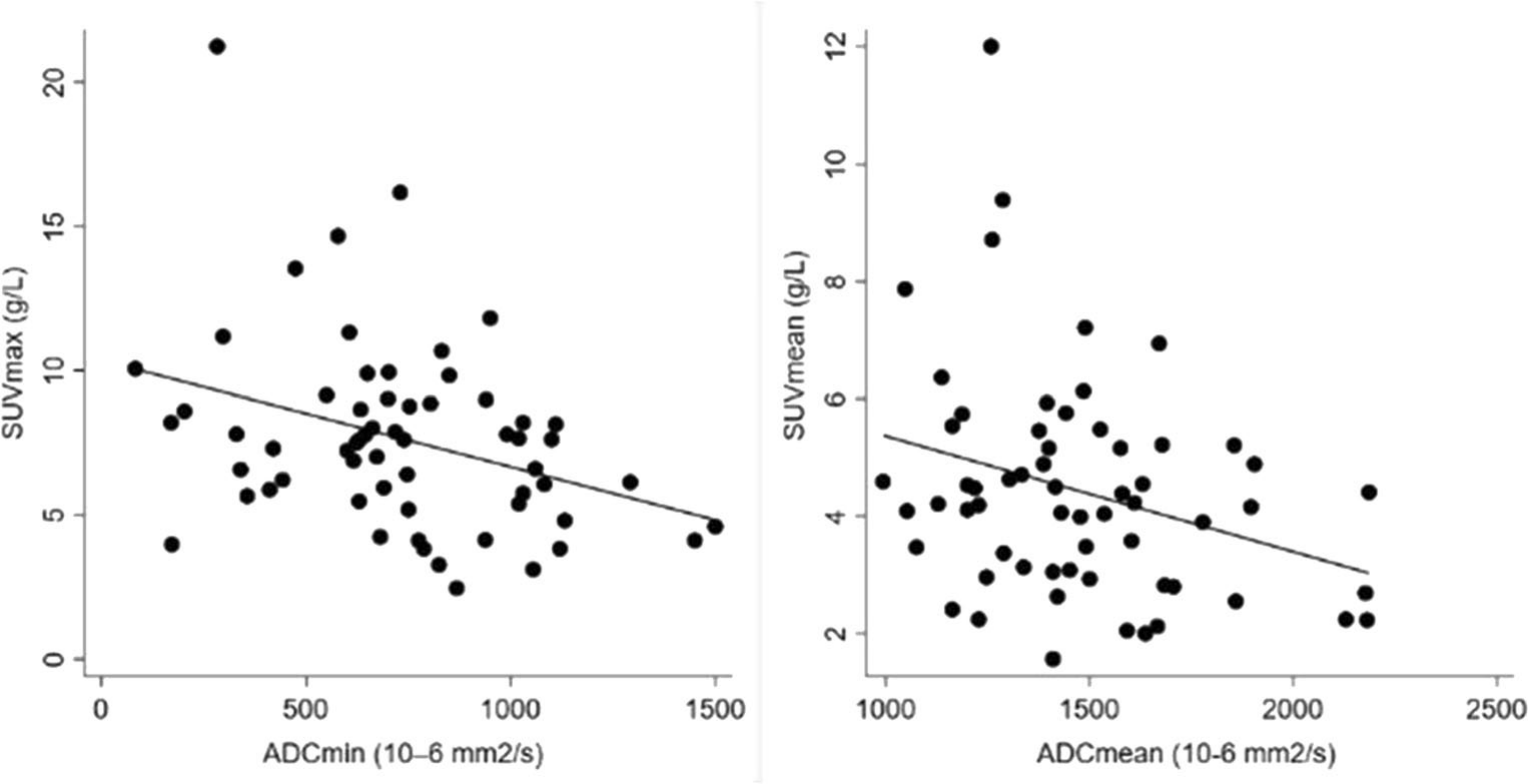
Correlation analysis between standardised uptake values (SUV in g/mL) and apparent diffusion coefficient (ADC in 10^–6^ mm^2^/s) values

**Fig. 3:**
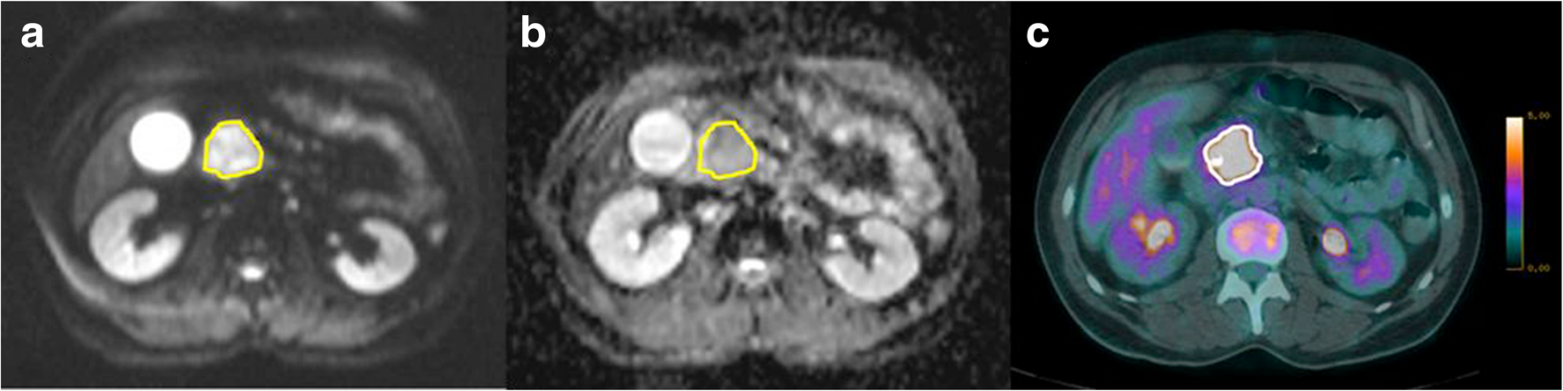
Example of a patient with untreated PDAC. Diffusion-weighted MRI (**a**, *b*-value: 800 s/mm^2^), ADC map (**b**), and PET/CT (**c**) were acquired in a 54-year-old male who underwent cephalic duodenopancreatectomy for a pT2N2M0 pancreatic ductal adenocarcinoma. The ADC_mean_, SUV_mean_, MTV, and MTV/ADC_min_ ratio were 1855 × 10^−6^ mm^2^/s, 5.2 g/mL, 20 mL, and 21 × 10^−3^, respectively

Quantitative parameters were similar between patients scanned on different PET scanners and at different MRI strength field (all *p* ≥ 0.15, Supplementary Table 4). We identified inverse correlations between SUV_max_ and ADC_min_ (rho = − 0.34, *p* = 0.0071) and between SUV_mean_ and ADC_mean_ (rho = − 0.29, *p* = 0.0026) (Figs. 2 and 3). ADC_min_ was inversely correlated with TLG (rho = − 0.40, *p* = 0.0015) and MTV (rho = − 0.28, *p* = 0.032), while ADC_mean_ was only significantly correlated with TLG (rho = − 0.30, *p* = 0.017). We detected a positive correlation between MTV and DTV (rho = 0.77, *p* < 0.0001). Only ADC_min_ values were inversely correlated with the tumour grade (rho = − 0.40, *p* = 0.0015) (Table 2).

**Table 2:** Correlation between imaging and pathological parameters in PDAC

	G	R	V	Pn	L
SUV_max_	0.20	− 0.13	0.04	− 0.24	0.01
SUV_mean_	0.25	− 0.12	0.05	− 0.24	0.02
MTV	− 0.02	0.15	0.15	− 0.02	− 0.03
TLG	0.07	0.04	0.14	− 0.07	0.01
ADC_min_	− 0.40*	0.05	0.06	0.09	0.05
ADC_mean_	− 0.24	0.07	− 0.05	− 0.02	− 0.04
DTV	− 0.03	0.26	0.05	− 0.13	− 0.13
MTV/ADC_min_	0.16	0.08	0.15	− 0.03	− 0.05

Values shown are Spearman’s rho values: *p = 0.0015, *p* > 0.05 for all other items. *ADC*, apparent diffusion coefficient; *DTV*, diffusion tumour volume; *G*, grade; *L*, lymphatic invasion; *MTV*, metabolic tumour volume; *PDAC*, pancreatic ductal adenocarcinoma; *Pn*, perineural invasion; *R*, quality of resection; *SUV*, standardised uptake value; *TLG*, total lesion glycolysis; *V*, vascular invasion

### Survival analysis

The median follow-up duration was 1.7 years (IQR: 0.8–2.3, range: 0.1–7.3 years) for the 41 patients included in the OS and PFS analyses and 1.7 years (IQR: 0.8–2.4 years, range: 0.3–7.3 years) for the 36 patients included in the DSS analysis. Only MTV had significant independent prognostic value for OS (Table 3) and for DSS (Table 4). DTV was an independent predictive factor for PFS (Table 5). OS, PFS, and DSS were not associated with the type of PET scanner or magnetic resonance strength field used (Tables 3, 4, and 5, all *p* ≥ 0.14).

**Table 3:** Predictive factors of OS (*n* = 41)

Variables	Univariate analysis			Stepwise multivariate analysis		
	HR	95% CI	*p* value	HR	95% CI	*p* value
Sex	0.87	0.46–1.62	0.65	—	—	—
1.5- vs. 3.0-T MRI	1.0	0.66–1.52	0.99	—	—	—
Discovery LS vs. 690	0.85	0.45–1.62	0.63	—	—	—
Stage 1–2 vs. 3–4	1.46	0.76–2.80	0.25	—	—	—
SUV_max_ > 7.3 (g/mL)	1.49	0.77–2.86	0.24	—	—	—
SUV_mean_ > 4.5 (g/mL)	1.22	0.65–2.33	0.53	—	—	–
MTV > 6.33 (cm^3^)	2.31	1.07–5.02	0.034	10.9	2.09–56.9	0.005
TLG > 24.77 (g·cm^3^/mL)	1.80	0.87–3.71	0.11	0.20	0.05–0.93	0.040
ADC_min_ > 939 (10^−6^ mm^2^/s)	0.79	0.39–1.60	0.51	—	—	—
ADC_mean_ > 1282 (10^−6^ mm^2^/s)	0.59	0.29–1.21	0.15	—	—	—
DTV > 4.72 (cm^3^)	2.27	0.98–5.26	0.056	—	—	—
MTV/ADC_min_ ratio > 8.31 (10^3^)	1.94	0.95–3.98	0.071	—	—	—

*ADC*, apparent diffusion coefficient; *DTV*, diffusion tumour volume; *MTV*, metabolic tumour volume; *OS*, overall survival; *PDAC*, pancreatic ductal adenocarcinoma; *SUV*, standardised uptake value; *TLG*, total lesion glycolysis. Bonferroni-corrected *p* value was 0.009 for MTV and 0.080 for TLG

**Table 4:** Predictive factors of DSS (*n* = 36)

Variables	Univariate analysis			Stepwise multivariate analysis		
	HR	95% CI	*p* value	HR	95% CI	*p* value
Sex	0.93	0.47–1.82	0.83	—	—	—
1.5- vs. 3.0-T MRI	0.99	0.64–1.56	0.99	—	—	—
Discovery LS vs. 690	0.77	0.38–1.54	0.45	—	—	—
Stage 1–2 vs. 3–4	1.56	0.77–3.16	0.22	—	—	—
SUV_max_ > 8.1 (g/mL)	1.28	0.64–2.54	0.49	—	—	—
SUV_mean_ > 4.5 (g/mL)	1.25	0.63–2.50	0.52	—	—	—
MTV > 6.33 (cm^3^)	2.63	1.10–6.27	0.029	12.8	2.15–76.0	0.005
TLG > 24.77 (g·cm^3^/mL)	2.27	0.99–5.18	0.052	—	—	—
ADC_min_ > 939 (10^−6^ mm^2^/s)	0.72	0.32–1.63	0.43	—	—	—
ADC_mean_ > 1282 (10^−6^ mm^2^/s)	0.59	0.27–1.27	0.18	—	—	—
DTV > 4.84 (cm^3^)	2.61	1.06–6.44	0.037	—	—	—
MTV/ADC_min_ ratio > 8.31 (10^3^)	1.93	0.87–4.26	0.10	0.20	0.04–1.0	0.049

*ADC*, apparent diffusion coefficient; *DSS*, disease-specific survival; *DTV*, diffusion tumour volume; *MTV*, metabolic tumour volume; *PDAC*, pancreatic ductal adenocarcinoma; *SUV*, standardised uptake value; *TLG*, total lesion glycolysis. Bonferroni-corrected *p* value was 0.010 for MTV and 0.099 for MTV/ADC_min_ ratio

**Table 5:** Predictive factors for PFS (*n* = 41)

Variables	Univariate analysis			Stepwise multivariate analysis		
	HR	95% CI	*p* value	HR	95% CI	*p* value
Sex	0.82	0.43–1.55	0.54	—	—	—
1.5- vs. 3.0-T MRI	0.85	0.55–1.30	0.45	—	—	—
Discovery LS vs. 690	0.62	0.33–1.17	0.14	—	—	—
Stage 1–2 vs. 3–4	1.47	0.90–2.40	0.13	—	—	—
SUV_max_ > 8.1 (g/mL)	1.64	0.84–3.20	0.15	—	—	—
SUV_mean_ > 4.49 (g/mL)	1.36	0.72–2.57	0.35	—	—	—
MTV > 6.33 (cm^3^)	3.11	1.34–7.23	0.008	—	—	—
TLG > 14.3 (g·cm^3^/mL)	6.53	1.50–28.34	0.012	—	—	—
ADC_min_ > 939 (10^−6^ mm^2^/s)	0.75	0.37–1.49	0.41	—	—	—
ADC_mean_ > 1274 (10^−6^ mm^2^/s)	0.88	0.45–1.74	0.72	—	—	—
DTV > 4.84 (cm^3^)	5.79	1.97–16.99	0.001	5.79	1.97–17.0	0.001
MTV/ADC_min_ ratio > 8.3 (10^3^)	2.44	1.18–5.03	0.016	—	—	—

*ADC*, apparent diffusion coefficient; *DTV*, diffusion tumour volume; *MTV*, metabolic tumour volume; *PDAC*, pancreatic ductal adenocarcinoma; *PFS*, progression-free survival; *SUV*, standardised uptake value; *TLG*, total lesion glycolysis

## Discussion

Our study yielded three main results. First, an inverse correlation between ADC and SUV was found, while only ADC_min_ was significantly correlated with tumour grade in PDAC patients. Second, MTV was a significant independent predictive factor for OS and DSS. Third, we demonstrated that DTV was an independent predictive factor for PFS. Overall, these results suggest that the combination of multiple PET-MRI metrics may help in the evaluation of tumour grade and prediction of PDAC patients’ survival.

Several prior studies have included radiopathological correlation analyses in patients with PDAC undergoing DW-MRI or ^18^F-FDG PET/CT, but few have included patients who underwent both examinations prior to surgery. To our knowledge, this is the first study to report a significant inverse correlation between ADC_min_ and tumour grade in PDAC. No significant correlation between ADC_mean_ or ^18^F-FDG PET–derived metrics and pathological parameters was observed.

Prior studies have reported discordant results regarding the relationship between ADC_min_ or ADC_mean_ and pathological findings. Chen et al [10] found a non-significant inverse association between ADC_min_ and tumour differentiation, and Kurosawa et al [24] reported that lower ADC_mean_ was associated with lower tumour differentiation. However, other authors [15, 25] have not described any significant association between ADC_mean_ and tumour grade. By nature, PDAC are fibrotic tumours; thus, some authors suggest that varying ADC values may be related to differences in fibrosis [26]. Nevertheless, this possibility is not supported by two recent studies [6, 15]. Furthermore, no correlation has been identified between ADC_mean_ and tumour microvessel or cell density [15]. Hayano et al [7] reported that tumours with low ADC_min_ exhibited a deeper invasion into the portal venous system and extrapancreatic nerve plexus compared with tumours with high ADC_min_. These conflicting results may be related to the heterogeneity of study populations, the variations in evaluated pathological parameters, and/or the different methods used for ADC measurement. Indeed, different authors have used single-ROI, multiple-ROI, or VOI, which can influence the reproducibility of ADC values [22]. Overall, the exact meaning of ADC values remains unclear and is likely related to an averaged multifactorial effect of tumour grade, cell density, and microenvironment.

Discordant results have also been published regarding the correlation between ^18^F-FDG uptake and proliferative activity. In a study including all histological subtypes of pancreatic cancers, Hu et al [4] reported a positive correlation between SUV_max_ and Ki-67, while Buck et al [14] found no statistical correlation. Ahn et al [13] detected a significant association between SUV_mean_ and tumour grade, while Im et al [8] reported no significant association between ^18^F-FDG parameters and tumour grade or perineural invasion, which agrees with the present results. However, Im et al also noted that TLG and MTV were marginally associated with lymphovascular invasion. Overall, these contradictory results suggest a crucial need for international standardisation of ADC measurements, and for large, multicentric studies, assessing the relationship between ^18^F-FDG PET- and DWI-derived parameters and pathological findings in resectable PDAC.

Several studies have demonstrated a significant correlation between ADC and SUV values in gastrointestinal tumours [18, 27–30]. However, few such studies have been performed in pancreatic cancer [10, 31]. In agreement with these results [10, 31], our present study showed significant inverse correlations between ADC_min_ and SUV_max_, and between ADC_mean_ and SUV_mean_ in patients with PDAC. Like Chen et al [10], we also detected a significant correlation between TLG and ADC_min_.

To date, few studies have examined the prognostic value of multiparametric PET-MRI. It remains unclear how PET and DWI parameters could be combined for use in the prognostic stratification of PDAC patients [26]. In a small sample of 17 patients operated for pancreatic ductal carcinoma, Chen et al [32] found better OS in patients with tumours exhibiting high ADC_min_ values compared to those with low ADC_min_ values. Similarly, Kurosawa et al [24] reported better OS in patients with tumours presenting high ADC_mean_ values than those with low ADC_mean_ values. Garces-Descovich et al [33] demonstrated that lower ADC values in PDAC were associated with worse 4-year OS. However, in agreement with Sakane et al [31], our findings showed no significant prognostic value for ADC_mean_ or ADC_min_.

We found that higher DTV predicted lower PFS, which had not previously been reported. Since DTV and MTV were collinear, this finding indicates that the tumour burden is a major prognostic biomarker.

Here, we demonstrated that MTV was a significant predicting factor for OS and DSS in operated PDAC patients. Several prior studies have also reported the prognostic value of ^18^F-FDG PET/CT–derived parameters [8, 9, 31, 34, 35]. However, these studies have shown high heterogeneity regarding the study population treatment (i.e., chemotherapy alone, chemoradiotherapy, surgery, or best supportive care) and the imaging protocol variables, making comparison difficult. In accordance with our present findings, Sakane et al [31] reported that SUV did not significantly predict OS. Also in agreement with our present results, two other studies [8, 9] found that MTV and TLG are independent predictors of OS and PFS in operated PDAC patients, regardless of neoadjuvant chemotherapy. Additionally, Chirindel et al [34] reported TLG as a predictive factor for PFS among unresectable PDAC patients. Interestingly, Hyun et al [35] showed that the intratumoural heterogeneity of ^18^F-FDG uptake (i.e., entropy) was an independent predictor of OS. However, their study population pooled patients treated with either surgery or various combinations of radiochemotherapy, which may deeply influence the outcome, such that these results cannot be not compared with our study. Finally, we found a trend for MTV/ADC_min_ ratio as a predictor of DSS, which is in agreement with results published by Chen et al from a smaller patients’ population [10]. Considering the inverse correlation between tumour grade and ADC_min_, this finding suggests that MTV/ADC_min_ ratio could reflect the impact of both tumour burden and grade on patient outcome.

Our study had several limitations. To our knowledge, this is the largest reported series with untreated and resected PDAC and with both preoperative DW-MRI and ^18^F-FDG PET/CT imaging; however, only 61 patients could ultimately be enrolled in the radiopathological correlation analysis, despite our long inclusion period of about 10 years. Furthermore, 20 of these 61 patients were lost for follow-up. Although this could limit the analysis power, we demonstrated that several PET-MR-derived parameters had significant prognostic value regarding OS, DSS, and PFS, which had not been previously performed before. Only one reader measured PET and MRI metrics. However, we used VOI methods that were proven the most reproducible to this purpose, suggesting limited impact. Moreover, several different PET and MRI scanners were used, but PET data stability was ensured by phantom acquisition over the study period. Also, we specifically compared PET and MRI data, as well as OS, PFS, and DSS according to scanners and found no significant difference, suggesting limited impact. Finally, the tumour measurements were always performed using the most reproducible available methods for ^18^F-FDG PET- and DWI-derived parameters, which overall suggests a limited impact on our results.

## Conclusions

In patients with resectable PDAC, tumour grade correlated with ADC_min_ values. MTV was predictive for OS and DSS, while DTV was predictive of PFS. These findings suggest that the combination of ^18^F-FDG PET- and DW-MRI-derived parameters may be more useful for prognostic stratification of PDAC patients, compared with single-modality imaging.

## Electronic supplementary material

ESM 1 (DOCX 28 kb)

## Abbreviations

^18^F: Fluorine-18
ADC: Apparent diffusion coefficient
CT: Computed tomography
DSS: Disease-specific survival
DTV: Diffusion tumour volume
DWI: Diffusion-weighted imaging
FDG: Fluorodeoxyglucose
IQR: Interquartile range
MRI: Magnetic resonance imaging
MTV: Metabolic tumour volume
OS: Overall survival
PDAC: Pancreatic ductal adenocarcinoma
PET: Positron emission tomography
PFS: Progression-free survival
PSF: Point source function
SUV: Standardised uptake value
TLG: Total lesion glycolysis
TOF: Time of flight
