# Supplementary material for: Prediction of tumour grade and survival outcome using pre-treatment PET- and MRI-derived imaging features in patients with resectable pancreatic ductal adenocarcinoma
Source: Eur Radiol. 2020 Aug 26;31(2):992–1001. doi: 10.1007/s00330-020-07191-z (PMC7813698; doi:10.1007/s00330-020-07191-z)
Supplement: Supplementary file 1 — (DOCX 28 kb) [file 330_2020_7191_MOESM1_ESM.docx]

**SUPPLEMENTARY MATERIALS**

**SUPPLEMENTARY TABLE 1.** TRIPOD Checklist : Prediction Model Development

| **Section/Topic** | **Item** | **Checklist Item** | **Page** |
| --- | --- | --- | --- |
| **Title and abstract** | | | |
| Title | 1 | Identify the study as developing and/or validating a multivariable prediction model, the target population, and the outcome to be predicted. | 1 |
| Abstract | 2 | Provide a summary of objectives, study design, setting, participants, sample size, predictors, outcome, statistical analysis, results, and conclusions. | 2 |
| **Introduction** | | | |
| Background  and objectives | 3a | Explain the medical context (including whether diagnostic or prognostic) and rationale for developing or validating the multivariable prediction model, including references to existing models. | 4-5 |
|  | 3b | Specify the objectives, including whether the study describes the development or validation of the model or both. | 5 |
| **Methods** | | | |
| Source of data | 4a | Describe the study design or source of data (e.g., randomized trial, cohort, or registry data), separately for the development and validation data sets, if applicable. | 6-9 |
|  | 4b | Specify the key study dates, including start of accrual; end of accrual; and, if applicable, end of follow-up. | 9 |
| Participants | 5a | Specify key elements of the study setting (e.g., primary care, secondary care, general population) including number and location of centres. | 6 |
|  | 5b | Describe eligibility criteria for participants. | 6 |
|  | 5c | Give details of treatments received, if relevant. | 6 |
| Outcome | 6a | Clearly define the outcome that is predicted by the prediction model, including how and when assessed. | 9 |
|  | 6b | Report any actions to blind assessment of the outcome to be predicted. | NA |
| Predictors | 7a | Clearly define all predictors used in developing or validating the multivariable prediction model, including how and when they were measured. | 8-9 |
|  | 7b | Report any actions to blind assessment of predictors for the outcome and other predictors. | NA |
| Sample size | 8 | Explain how the study size was arrived at. | 6 |
| Missing data | 9 | Describe how missing data were handled (e.g., complete-case analysis, single imputation, multiple imputation) with details of any imputation method. | 9 |
| Statistical analysis methods | 10a | Describe how predictors were handled in the analyses. | 10 |
|  | 10b | Specify type of model, all model-building procedures (including any predictor selection), and method for internal validation. | 10 |
|  | 10c | Specify all measures used to assess model performance and, if relevant, to compare multiple models. | NA |
| Risk groups | 11 | Provide details on how risk groups were created, if done. | 10 |
| **Results** | | | |
| Participants | 13a | Describe the flow of participants through the study, including the number of participants with and without the outcome and, if applicable, a summary of the follow-up time. A diagram may be helpful. | 11 |
|  | 13b | Describe the characteristics of the participants (basic demographics, clinical features, available predictors), including the number of participants with missing data for predictors and outcome. | 11 |
| Model development | 14a | Specify the number of participants and outcome events in each analysis. | 12 |
|  | 14b | If done, report the unadjusted association between each candidate predictor and outcome. | 12 |
| Model specification | 15a | Present the full prediction model to allow predictions for individuals (i.e., all regression coefficients, and model intercept or baseline survival at a given time point). | Tables |
|  | 15b | Explain how to the use the prediction model. | NA |
| Model performance | 16 | Report performance measures (with CIs) for the prediction model. | NA |
| **Discussion** | | | |
| Limitations | 18 | Discuss any limitations of the study (such as nonrepresentative sample, few events per predictor, missing data). | 16 |
| Interpretation | 19 | Give an overall interpretation of the results, considering objectives, limitations, and results from similar studies, and other relevant evidence. | 13-16 |
| Implications | 20 | Discuss the potential clinical use of the model and implications for future research. | 13-16 |
| **Other information** | | | |
| Supplementary information | 21 | Provide information about the availability of supplementary resources, such as study protocol, Web calculator, and data sets. | NA |
| Funding | 22 | Give the source of funding and the role of the funders for the present study. | NA |

**SUPPLEMENTARY TABLE 2.** Technical Parameters of PET

|  | **Discovery LS** | **Discovery 690** |
| --- | --- | --- |
| **PET Acquisition** |  |  |
| Time per bed position | 3-5 min | 2 min |
| Collimation | 2D with septa | 3D |
| **PET Reconstruction** |  |  |
| Algorithm | OSEM | OSEM |
| Iterations | 2 | 3 |
| Subsets | 28 | 16 |
| Post filter | 5.4 FWHM | 5.0 FWHM |
| Loop filter | 3.91 FWHM | No |
| Time-of-flight correction | No | Yes (500 ps) |
| Point spread function correction | No | Yes |
| Slice thickness | 4.2 mm | 3.3 mm |
| Diameter | 50 cm | 70 cm |
| Matrix | 128 x 128 | 256 x 256 |
| Voxel size | 4.2 x 3.9 x 3.9 mm | 3.3 x 2.7 x 2.7 mm |

FWHM full width at half maximum, PSF: point spread function, OSEM: ordered subset expectation maximization, TOF: time-of-flight

**SUPPLEMENTARY TABLE 3.** Technical Parameters of MR Sequences

| **Sequences** | **Imaging**  **plane** | **TE/TR,**  **msec** | **Flip**  **angle** | **Pixel**  **Size, mm** | **NEX** | **Slice thickness, mm** | **Parallel**  **Imaging**  **(iPAD)** | **Respiratory**  **method** |
| --- | --- | --- | --- | --- | --- | --- | --- | --- |
| **HASTE** | Axial | 88–96/  1500–1600 | 160° | 1.3 × 1.3 | 1 | 3 | GRAPPA | Breath  hold |
| **HASTE** | Coronal | 87–101/  1200–1800 | 160° | 1.3–1.6 ×  1.3–1.6 | 1 | 3–4 | GRAPPA | Breath  hold |
| **Diffusion SPAIR** | Axial | 63–66/  3700–7700 | 90° | 1.4–3.5 ×  1-4–2.8 | 1-6 | 5–6 | GRAPPA | Free  Breathing |
| **3D-VIBE** | Axial | 1.3–4.5/  3.97–6.68 | 9–10° | 1.2–1.8 ×  1.2–1.5 | 1 | 3 | GRAPPA,  CAIPIRINHA | Breath hold |
| **3D-VIBE** | Coronal | 1.3–4.5/  4.2–6.68 | 9–13° | 1.4–1.9 ×  1.4–1.8 | 1 | 3 | GRAPPA,  CAIPIRINHA | Breath hold |

HASTE indicates half-Fourier acquisition single-shot turbo-spin-echo; SPAIR, spectral adiabatic inversion recovery fat suppression; 3D-VIBE, three-dimensional volumetric interpolated breath hold examination; iPAD, integrated parallel acquisition technique; GRAPPA, generalized auto-calibrating partially parallel acquisitions; and CAIPRINHA, controlled aliasing in parallel imaging results in higher acceleration.

**SUPPLEMENTARY TABLE 4.** Pre-treatment PET and MRI derived metrics comparison between different PET and MRI scanners.

| **Variables** | **Discovery LS**  **n=34** | **Discovery 690**  **N=27** | ***P* value** |
| --- | --- | --- | --- |
| **SUV_max_** | 8.0 ± 2.5 | 7.2 ± 4.1 | 0.35 |
| **SUV_mean_** | 4.7 ± 1.4 | 4.1 ± 2.3 | 0.21 |
| **MTV** | 12.8 ± 10.1 | 14.9 ± 11.6 | 0.45 |
| **TLG** | 59.7 ± 47.1 | 63.3 ± 64.5 | 0.80 |
|  | **1.5 Tesla MRI**  **n=33** | **3.0 Tesla MRI**  **n=28** |  |
| **ADC_min_** | 799 ± 294 | 697 ± 268 | 0.17 |
| **ADC_mean_** | 1503 ± 286 | 1404 ± 237 | 0.15 |
| **DTV** | 12.5 ± 8.6 | 13.8 ± 12.6 | 0.64 |

Mean ± standard deviation are presented. ADC indicates apparent diffusion coefficient; DTV, diffusion tumor volume; MTV, metabolic tumor volume; SUV, standardized uptake value; and TLG, total lesion glycolysis.
